# Calcium-Dependent Protein Kinases in Plants: Structure, Signaling, and Multifaceted Regulatory Roles in Development and Stress Adaptation

**DOI:** 10.3390/ijms27041843

**Published:** 2026-02-14

**Authors:** Rui Wang, Jiangyu Meng, Shuwen Yang, Bingjie Sun, Wei Qian, Yajun He

**Affiliations:** 1 Academy of Agricultural Sciences, Southwest University, Chongqing 400715, China; wr20000818@163.com (R.W.); 13002363008@163.com (J.M.);; 2 Integrative Science Center of Germplasm Creation in Western China (Chongqing) Science City, College of Agronomy and Biotechnology, Southwest University, Chongqing 400715, China; 3 Engineering Research Center of South Upland Agriculture, Ministry of Education, Chongqing 400715, China

**Keywords:** calcium-dependent protein kinases, growth and development, stress adaptation

## Abstract

Calcium-dependent protein kinases (CDPKs) are crucial calcium sensors in plants that perceive transient calcium fluctuations. Structurally, CDPKs contain an integrated serine/threonine protein kinase domain, which enables them to function as protein kinases. Through phosphorylation of downstream target proteins, CDPKs transduce specific calcium-encoded signals to regulate diverse physiological processes. As pivotal signaling molecules, CDPKs function as critical regulators in plant growth, development, and stress responses. This review comprehensively summarizes the structure of CDPKs and their signal transduction mechanisms. It further elaborates on the multifaceted functions of CDPKs across diverse plant species, encompassing their regulatory roles in developmental processes, responses to abiotic and biotic stresses, and intricate interactions with phytohormone signaling pathways.

## 1. Introduction

The morphogenesis and physiological development of plants are coordinately regulated by endogenous genetic factors and exogenous environmental signals. The detection and conversion of external signals by cells result in the reprogramming of gene transcription, which eventually initiates adaptive physiological and biochemical responses. In eukaryotic cell signaling, calcium ions (Ca^2+^), which are essential second messengers, play crucial roles. Calcium ions integrate signaling cascades triggered by multiple environmental and endogenous cues, including but not limited to photostimulation, thermic variations (both hyperthermic and hypothermic conditions), mechanostimulation (thigmotropism), ionic imbalance (Na^+^ hyperaccumulation), hydric limitation, osmolarity shifts, phytohormonal regulation, mycological elicitation, and rhizobial symbiosis initiation signals [1,2].

**Figure 1:**
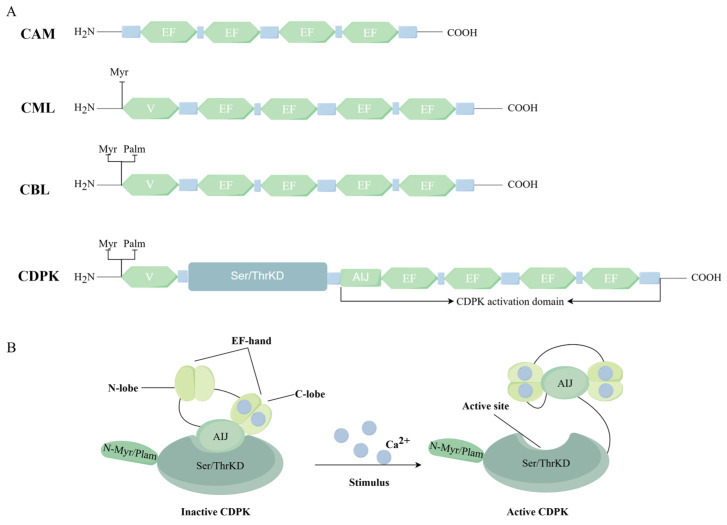
Typical Structure of Ca^2+^-Sensor Proteins and the Domain Architecture and Activation Mechanism of CDPK Proteins. (**A**) Typical domain architecture of Ca^2+^-Sensor Proteins in plant. Calmodulin (CaM), CaM-like proteins (CMLs), calcineurin B-like proteins (CBLs), and CDPKs all possess Ca^2+^-binding EF-hand motifs. CDPKs further include a Ser/Thr kinase domain and a CDPK activation domain (CAD); the latter is made up of an autoinhibitory junction (AIJ) and a CaM-like domain that carries EF-hands. (**B**) The relief-of-autoinhibition model for plant CDPK activation, proposed by Harmon et al. (1994), Harper et al. (1994), and Yoo and Harmon (1996) [17,18,19]. When [Ca^2+^]cyt is low, Ca^2+^ binds to the C-terminal EF-hand lobe, and the CAD then blocks the kinase’s active site. As [Ca^2+^]cyt rises, Ca^2+^ can bind the N-terminal EF-lobe; this interaction triggers an allosteric change that pulls the CAD away from the kinase’s active site, ultimately promoting the protein’s active conformation. By www.figdraw.com (accessed on 9 December 2025).

Upon stimulation, cells undergo alterations in the concentration of cytosolic Ca^2+^ ([Ca^2+^]_n_) due to the stimulus, generating distinct “calcium signatures” that are spatiotemporally encoded at the cellular level. These stimulus-specific [Ca^2+^]_n_ dynamics exhibit both cell-type specificity and spatial heterogeneity across tissue regions, reflecting the precise regulation of calcium-dependent signaling cascades [3,4,5,6]. Plants rely on a diverse array of calcium-sensing proteins—including calmodulin (CaM/CAM), CaM-like proteins (CMLs), calcium-dependent protein kinases (CDPKs/CPKs), calcineurin B-like proteins (CBLs), and CBL-interacting protein kinases—to perceive and transduce stimulus-specific calcium signals to downstream effectors [7,8,9,10]. These four classes of proteins function as calcium signal transducers, all of which contain conserved calcium-binding structures called EF-hand domains. The canonical EF-hand domain comprises 29 amino acid residues that form a helix–loop–helix (HLH) structural motif, with 12 central residues constituting the high-affinity calcium-binding site [11]. Upon Ca^2+^ binding, these Ca^2+^-sensor proteins undergo conformational rearrangements, enabling them to allosterically regulate their target proteins and transmit upstream signals to downstream effectors [12,13,14,15]. CDPKs are distinct from the other three calcium sensors because they uniquely incorporate an EF-hand domain for Ca^2+^ detection and a functional serine/threonine kinase domain that is regulated by Ca^2+^ (Figure 1A). This dual-domain architecture enables CDPKs to directly transduce Ca^2+^ signals through substrate phosphorylation [16].

## 2. Structures of CDPKs

CDPKs are widely present in plants, algae, protists, and some fungi. They consist of a serine/threonine kinase domain and a CDPK activation domain (CAD), with the latter comprising an autoinhibitory junction (AIJ) and a calmodulin-like domain that contains an EF-hand motif (Figure 1A). The last century proposed the ‘autoinhibition release model’ for plant CDPK activation by Harmon et al. (1994), Harper et al. (1994), and Yoo and Harmon (1996). At low cytoplasmic Ca^2+^ levels, Ca^2+^ attaches to the C-terminal EF-hand lobe, leading the CAD to obstruct the kinase’s active site and keep the protein inactive. When [Ca^2+^]cyt increases, Ca^2+^ binds with the N-terminal EF-hand lobe, which triggers an allosteric change in its conformation. CAD is displaced from the kinase active site by this change, which in turn supports the formation of an active kinase conformation (Figure 1B) [17,18,19].

In the 21st century, research on the structure and activation mechanisms of CDPKs has made significant progress. Chandran et al. (2006) employed X-ray crystallography to determine the crystal structure of the J-CaM-LD domain in AtCPK1. They revealed the structural details of the interaction between the CaM-LD and J domains. These findings provide experimental support for the structural model of the intramolecular J-CaM-LD complex in kinase activation [20]. The active states of apicomplexan CDPKs bound to Ca^2+^, including *Cryptosporidium parvum* CpCDPK1/3 and *Toxoplasma gondii* TgCDPK1, were determined in crystal structures by Wernimont et al. (2011) [15]. Additionally, they resolved the Ca^2+^-unbound inactive-state crystal structures of TgCDPK1 and TgCDPK3. These architectures of parasitic CDPK domains indicate a shared activation mechanism. Dixit and colleagues were able to identify two distinct sites on CDPK that bind calcium. Their analysis demonstrated that the tertiary structure of CDPK changes sequentially as calcium binds. By observing heat exchange, they quantitatively demonstrated that calcium binding results in modifications to both the secondary and tertiary structures of CaCDPK1 [21]. As demonstrated by numerous studies, the N-terminal variable domain of CDPK contains subcellular targeting information [22,23,24,25].

## 3. CDPKs: Key Integrators Bridging Organ Development and Reproductive Transition in Plants

CDPKs function as key signaling molecules in plants and exhibit broad tissue expression patterns. They are detectable in various vegetative organs, and play crucial roles in multiple stages of both reproductive and vegetative development, thereby significantly contributing to the orderly progression of the plant life cycle [26]. During vegetative growth, CDPKs exert regulatory functions primarily through phytohormone signaling pathways [27,28,29,30]. By phosphorylating downstream components, they precisely modulate the transduction of auxin, cytokinin, and other hormonal signals, thereby influencing cell proliferation, tissue differentiation, and organ morphogenesis to ensure normal growth and development [31,32,33]. In the reproductive phase, CDPKs display multiple functions. On the one hand, they are directly involved in pollen tube elongation through the modulation of cytoskeletal organization and Ca^2+^ gradients and support pollen viability, thereby ensuring effective fertilization. On the other hand, CDPKs function as central integrators linking environmental signals, such as photoperiod and temperature, to internal flowering regulatory networks. These regulatory networks encompass specific pathways, such as florigen signaling. This integration allows CDPKs to indirectly regulate flowering time and ensure the precise regulation of reproductive transition [34,35,36].

### 3.1. CDPK in Pollen Tube Growth

**Figure 2:**
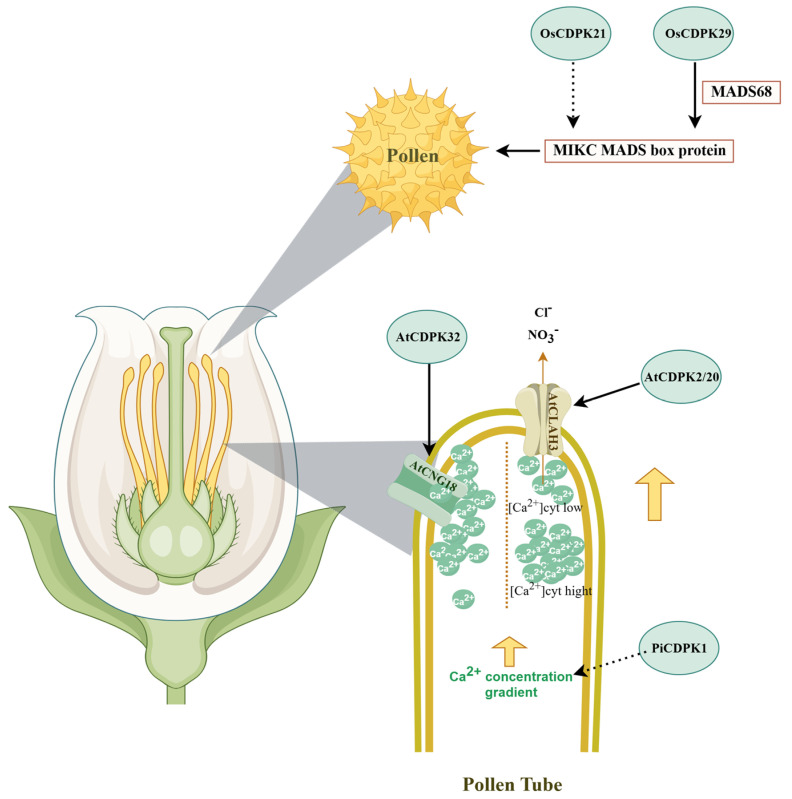
CDPK regulates pollen tube growth and pollen development through multiple pathways. PiCDPK1 is potentially involved in regulating pollen tube growth, likely through the maintenance of Ca^2+^ homeostasis [37]. AtCDPK32 facilitates Ca^2+^ influx into the pollen tube by activating the channel AtCNGC18 [38]. AtCDPK2 and AtCDPK20 phosphorylate the anion channel SLAH3 to regulate pollen tube growth [39]. OsCDPK21 likely participates in pollen development by modulating the transcription of OsMADS63 and OsMADS68 [40]. OsCDPK29 is involved in regulating pollen growth and directly interacts with OsMADS68 to modulate its transcriptional activity [41]. By www.figdraw.com (accessed on 9 December 2025).

Ca^2+^ is essential for controlling the polarized growth of pollen tubes, which are the tip-growing cells in plant. In multiple species, CDPKS have been found to be involved in the regulation of pollen tube growth by translating Ca^2+^ signals into specific phosphorylation events that orchestrate cytoskeletal dynamics, vesicle trafficking, and apical growth (Figure 2).

In petunia (*Petunia hybrida*), PiCDPK1 has been demonstrated to modulate pollen tube elongation, highlighting the functional relevance of CDPK-mediated signaling in tip-growing cells [37]. In maize, ZmCDPK32 acts as a suppressor of pollen tube elongation [38]. In *Arabidopsis thaliana*, CDPK17 and CDPK34 play essential roles in the apical growth of pollen tubes, serving as key molecular hubs that integrate calcium signaling with the regulation of apical development and tropic responses [42]. AtCDPK32 plays a crucial role in maintaining pollen tube growth polarity and facilitating an increase in cytoplasmic Ca^2+^ levels at the tube apex. This phenotype is likely mediated through physical and functional interactions with cyclic nucleotide-gated channel 18 (CNGC18), which modulates calcium influx and spatial distribution [43,44]. The growth of pollen tube is highly sensitive to ion fluxes, requiring precise regulation of ion dynamics to maintain directional expansion and cellular integrity [45]. CDPK11 and CDPK24 modulate the function of inward-rectifying potassium (K^+^) channels, thereby contributing to the control of pollen tube growth [46]. CDPK2 and CDPK20 promote pollen tube growth by phosphorylating and regulating the anion channel SLAH3 in a calcium-dependent manner [39]. These findings demonstrate that CDPKs regulate pollen tube elongation and polar growth through multiple pathways. However, in both reported and unexamined species, the precise regulatory networks involved remain to be further elucidated. The activity and regulatory functions of CDPKs are directly modulated by Ca^2+^ levels. Therefore, investigating the role of these calcium-responsive kinases in pollen tube development represents a significant and promising research direction.

### 3.2. CDPK in Pollen Development

Pollen is crucial in the sexual reproduction of flowering plants. Its development is a complex process involving numerous enzymes, transcription factors, and signaling molecules (Figure 2). In rice, several CDPKs have been implicated in regulating pollen development. Among them, CDPK9 acts as a positive regulator of pollen viability and spikelet fertility [47]. MADS-box genes encode transcription factors that serve as master regulators of pollen maturation [48]. OsCDPK21 likely participates in late-stage pollen development by modulating the transcription of *OsMADS63* and *OsMADS68* [40]. OsCDPK29 is also involved in regulating pollen growth. It directly interacts with OsMADS68 to modulate its transcriptional activity, thus governing the transcription of genes critical for pollen maturation [41]. The functional characterization of CDPKs during pollen development and maturation remains largely unexplored in many species. However, studies in rice indicate that the CDPK family participates in regulating late pollen development and influences pollen viability. This process involves multiple distinct CDPK genes, and two or more CDPK proteins may operate within the same regulatory pathway. Whether their functions are synergistic, antagonistic, or independent represents an open question worthy of further investigation.

### 3.3. CDPK in Seed Development

CDPKs play regulatory roles in seed development across diverse crop species. Early studies indicated that *OsCDPK2* influences seed development [49]. *OsCDPK1* suppresses amylose accumulation, enhances endosperm transparency, and reduces seed dimensions during grain development [50]. *OsCDPK31* is proposed to interact with genes involved in starch accumulation and grain filling. Unlike *OsCDPK1*, which influences starch composition in seeds, *OsCDPK31* modulates the timing of seed maturation and starch accumulation without affecting final seed size [51]. During castor bean (*Ricinus communis*) seed development, CDPKs participate in the regulation of carbohydrate metabolism. Specifically, *Rc*CDPK2 phosphorylates *Rc*SUS1, thereby modulating the production of UDP-glucose for the biosynthesis of storage compounds [52]. Moreover, RcCDPK1 mediates inhibition of the bacterial-type PEPC subunit via phosphorylation within a unique class 2 heteromeric PEPC complex during castor seed development, thereby modulating storage oil and protein biosynthesis [53,54]. CDPKs play regulatory roles in substrate accumulation during seed development, with their functions varying across different pathways and developmental stages even within the same species. Moreover, as signaling proteins, they also participate in controlling seed dormancy and germination by modulating hormone signaling pathways. AtCDPK12 suppresses ABA signaling throughout seed germination and early seedling development [55,56]. TaCDPK40 modulates ABA sensitivity, negatively regulating dormancy while positively promoting germination in wheat during seed germination [57].

### 3.4. CDPK in Root Growth

Root hair cells, as tip-growing structures, exhibit growth that is strongly influenced by Ca^2+^. Analogous to their role in pollen tube growth regulation, CDPKs modulate root hair growth by regulating calcium ion concentrations, a mechanism well documented in *Arabidopsis thaliana*. CDPK1 drives root hair development by phosphorylating and modulating the activity of the cyclic nucleotide-gated channels CNGC5, CNGC6, and CNGC9, thereby fine-tuning the cytosolic Ca^2+^ concentration to support sustained apical growth [58]. Glutamate receptor-like (GLR) proteins mediate Ca^2+^ influx in plants. Among them, GLR3.6 is involved in root growth, and CDPK16 specifically phosphorylates GLR3.6 at the Serine-856 residue, which is essential for its function in regulating root development [59,60]. Overexpression of a constitutively active variant of CDPK30 inhibits root growth and leads to aberrant auxin accumulation at the root tip in *Arabidopsis thaliana*. This phenotypic alteration may result from CDPK30-mediated modulation of PIN protein levels, thereby disrupting polar auxin transport and ultimately impairing root development [61].

## 4. CDPKs: Pioneering Signaling Mediators in Plant Abiotic Stress Transduction Amid Environmental Stimuli

Plants, which grow without movement, are frequently challenged by a range of abiotic and biotic stresses. These stresses significantly impair plant growth and productivity. However, upon detection of environmental stimuli, plants initiate intracellular signal transduction cascades to regulate gene expression and trigger appropriate physiological and biochemical responses. CDPKs function as essential signaling components mediating plant responses to diverse abiotic stresses.

### 4.1. CDPK Under Drought Stress

**Figure 3:**
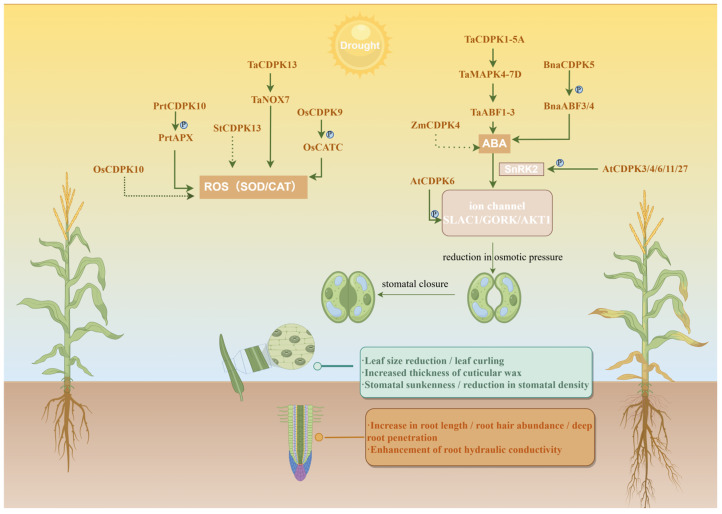
CDPKs modulate drought tolerance across different plant species. ZmCDPK4 is likely involved in plant drought tolerance through the ABA signaling pathway [69]. TaCDPK1-5A interacts with TaMAPK4-7D to regulate TaABF1-3, thereby modulating ABA signaling and positively conferring drought stress tolerance [74]. AtCDPK3, 4, 6, 11, and 27 phosphorylate SnRK2s to modulate ion channel activity and regulate stomatal movement [84]. BnaCDPK5 phosphorylates BnaABF3/4, thereby participating in the ABA signaling pathway [75]. AtCDPK6 phosphorylates SLAC1, thereby regulating ion flux and ultimately promoting stomatal closure [79]. PtrCDPK10 binds to and phosphorylates PtrAPX, thereby enhancing ROS detoxification [88]. StCDPK13 functions through the modulation of peroxidase activity to contribute to drought tolerance in plants [89]. OsCDPK10 is implicated in the drought stress response, potentially through its interaction with peroxidases [91]. OsCDPK9 enhances drought tolerance by phosphorylating CATC [90]. TaCDPK13 directly interacts with TaNOX to modulate the generation of ROS in wheat [92]. By www.figdraw.com (accessed on 9 December 2025).

Drought is a primary abiotic constraint that severely impairs plant growth and development. Under drought stress, cellular dehydration leads to reduced turgor pressure and inhibited cell wall expansion, thereby critically compromising plant growth and structural integrity. Drought results in water deficit within plants, disrupting the normal stomatal closure rhythm. Plants induce stomatal closure as a water conservation strategy; however, this modification in stomatal conductance results in reduced photosynthetic activity. As a result, energy synthesis is inhibited, often culminating in metabolic disruption and the buildup of toxic substances [62,63]. Plants employ three primary strategic responses to drought stress: escape, avoidance, and tolerance. Drought escape involves modifications in the life cycle to complete reproduction before severe stress occurs. Drought avoidance is characterized by the prevention of tissue water loss, often through mechanisms that reduce soil water depletion, which is primarily regulated by stomatal responses. Abscisic acid (ABA) is essential for controlling the stomatal aperture through its influence on guard cell behavior [64]. Under drought conditions, metabolic imbalance in plants severely impairs growth performance and can lead to oxidative damage [65,66,67]. CDPKs are extensively documented as key regulators of drought resistance in numerous plant species, such as *Arabidopsis thaliana*, maize (*Zea mays*), rice (*Oryza sativa*), and sorghum (*Sorghum bicolor*) [68,69,70,71,72]. Research indicates that CDPKs modulate plant responses to drought via diverse molecular pathways, ultimately affecting drought adaptation (Figure 3). Specific CDPK isoforms are implicated in ABA signal transduction, playing a functional role in enhancing plant resilience under water deficit. Under conditions of osmotic stress, ZmCDPK4 mediates drought adaptation in maize through the upregulation of key transcription factors such as ABF3, ABI5, and RAB18 [73]. TaCDPK1-5A can interact with TaMAPK4-7D, and TaMAPK4-7D subsequently associates with TaABF1-3. This protein interaction module modulates the sensitivity of ABA signaling and positively regulates drought stress tolerance [74]. In *Brassica napus*, BnaCDPK5 phosphorylates BnaABF3 and BnaABF4. This phosphorylation enhances the upregulation of the drought-responsive marker gene responsive to dehydration (RD) 29B. Accordingly, BnaCDPK5 enhances drought tolerance in this species [75]. Under drought conditions, ABA induces anion and potassium efflux from guard cells via plasma membrane ion channels. This ionic loss decreases cellular turgor, thereby inducing stomatal closure to reduce transpiration [62,76,77]. Research across multiple plant species has confirmed the involvement of CDPKs in this regulatory mechanism. Specifically, AtCDPK23 of Arabidopsis thaliana contributes to stomatal movement control through the modulation of potassium (K^+^) uptake [78]. *AtCDPK6* strongly activates the anion channel SLAC1 through phosphorylation; this channel performs an essential function in facilitating stomatal closure [79]. Stomatal closure induction and stomatal opening inhibition mediated by ABA and Ca^2+^ are compromised in *cpk10* mutants [80]. To mitigate drought stress, *AtCDPK8* contributes to ABA-mediated stomatal regulation by modulating the activity of CATALASE3 (CAT3) [81]. ZmCPK35 and ZmCPK37 mediate ABA-triggered stomatal closure through the activation of ZmSLAC1. Similarly, OsCPK9 in rice contributes to drought tolerance by promoting stomatal closure and improving osmotic adjustment [47,82]. SnRK2 kinases play a pivotal role in the ABA signaling cascade, which is governed by ABA receptors (PYR/PYL/RCAR) and class A protein phosphatases 2C (PP2Cs). During ABA-induced stomatal closure, the Arabidopsis guard cell-predominant inward-rectifying K^+^ channel KAT1 is functionally regulated via phosphorylation, which is mediated by the key ABA signaling kinase SnRK2.6/OST1 [83]. AtCDPK3, 4, 6, 11, and 27 were identified by Li et al. (2025) as kinases that respond rapidly to osmotic stress. Moreover, these CDPKs interact with and phosphorylate SnRK2s, thereby contributing to the regulation of drought tolerance [84].

Water deficit in plants triggers cellular homeostasis disruption and results in reactive oxygen species (ROS) accumulation. Overproduction of ROS may have harmful effects on vital cellular structures [85,86]. Consequently, maintaining ROS levels within subtoxic concentrations is crucial for preventing oxidative damage under stress conditions [87]. In trifoliate orange (*Poncirus trifoliata* (L.) Raf.), PtrCDPK10 binds to and phosphorylates ascorbate peroxidase (PtrAPX), thereby enhancing ROS detoxification. This mechanism contributes to ROS homeostasis and improves both dehydration and drought tolerance [88]. In potato, transgenic overexpression of StCDPK13 significantly increases antioxidant enzyme activities under drought conditions. Moreover, hydrogen peroxide (H_2_O_2_) levels were significantly decreased. These findings indicate that StCDPK13 overexpression strengthens ROS detoxification and mitigates oxidative stress caused by drought [89]. OsCDPK10 facilitates catalase protein accumulation, which helps reduce oxidative injury during drought. Specifically, OsCDPK9 targets catalase C (OsCATC) for phosphorylation at the plasma membrane. This modification enhances the enzymatic function of OsCATC, promoting the elimination of ROS and increasing plant drought resistance [90,91]. CDPKs not only regulate peroxidase-mediated ROS scavenging but are also involved in modulating oxidase-dependent ROS synthesis, thereby contributing to the fine-tuning of ROS homeostasis. In wheat, TaCDPK13 associates with TaNOX7, an NADPH oxidase, to regulate ROS generation. This protein interaction is essential for developmental processes and drought resistance in wheat [92].

### 4.2. CDPK Under Salinity Stress

Salt stress is a critical environmental factor that severely impairs plant growth and development; it disrupts metabolic processes, leading to cellular dysfunction, inhibition of photosynthesis, and nutrient imbalance [93]. To survive in saline environments, plants have developed many physiological and biochemical mechanisms. These include increased Na^+^ exclusion, restricted Na^+^ uptake, the regulation of cellular ion homeostasis, the activation of antioxidant enzyme systems to modulate ROS levels, and the modulation of phytohormone signaling to improve salinity adaptation [94,95].

Multiple studies have demonstrated that in rice, *OsCDPK4*, *OsCDPK5*, *OsCDPK7*, *OsCDPK12*, *OsCDPK13*, and *OsCDPK21* promote salinity stress resistance. Among these, OsCDPK5 and OsCDPK13 improve salt tolerance by coordinately regulating ROS production and detoxification pathways [96,97,98,99,100]. Mitogen-activated protein kinases (MAPKs), where OsMPK3/6 function as key positive regulators, are central during salt stress signaling. According to Su et al. (2024), *OsCDPK5*/*13* facilitates the activation of OsMPK3/6 through phosphorylation to increase salt tolerance. This defines an MKK-independent, atypical MAPK pathway that plays a major role in promoting salinity tolerance in rice [99]. By phosphorylating the vacuolar K^+^ channel TPK1 under salt stress, AtCDPK3 promotes sustained K^+^ efflux from the vacuole to the cytoplasm in Arabidopsis. This process restores the cytosolic K^+^/Na^+^ ratio, thereby improving plant resilience to sodium-specific salinity [101]. In wheat (*Triticum aestivum*), *TaCDPK5* and *TaCDPK9-1* are activated under salt stress. These kinases interact with and phosphorylate TaPP2CA116/12 and TabZIP60, potentially acting as a molecular link connecting calcium-mediated signals and ABA-regulated pathways. The PP2CA–CDPK–ABF/AREB module thus contributes to the regulation of salt tolerance [102]. DREB (dehydration-responsive element-binding) proteins, functioning as central regulators, predominantly mediate ABA-independent regulation of stress-responsive genes. In pumpkin, CmoCDPK20 phosphorylates CmoDREB2A, enabling it to bind more strongly to the promoter of the jasmonic acid (JA) biosynthetic gene CmoAOC2. This phosphorylation enhances jasmonic acid (JA) biosynthesis, increases peroxidase (POD) and catalase (CAT) activities, and consequently improves salinity resilience in grafted cucumber [103,104]. Polyamines (PAs), low-molecular-weight aliphatic nitrogenous alkaloids with notable biological activity, positively regulate plant salt tolerance by mediating biosynthesis and signaling pathways that modulate antioxidant enzymes [105]. S-Adenosylmethionine (SAM) functions as a biosynthetic precursor for PA synthesis in plant cells [106]. In cucumber (Cucurbita sativus), CsCDPK6 and CsSAMS1 modulate ethylene metabolism and PA conversion. CsCDPK6 overexpression confers salt tolerance in tobacco by regulating ROS levels, stomatal density, and stomatal aperture under salt stress conditions [107].

### 4.3. CDPK Under Cold Stress

**Figure 4:**
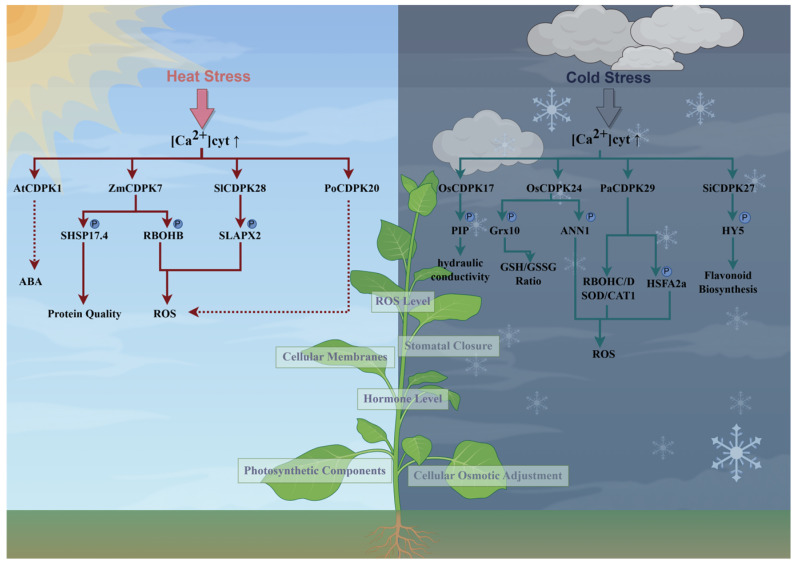
CDPKs are involved in mediating plant responses to low-temperature and high-temperature stress. OsCDPK17 phosphorylates and stimulates the aquaporin OsPIP2;1, OsPIP2;6; thereby enhancing cellular osmoregulatory capacity [111]. OsCDPK24 phosphorylates OsGrx10, leading to elevated glutathione levels and an increased GSH/GSSG ratio; OsCDPK24 phosphorylates OsANN1 to regulate ROS homeostasis [112]. PpCDPK29 functions by interacting with key components of ROS metabolism (PpRBOHC/D, PpSOD, PpCAT1) and by phosphorylating the transcription factor PpHSFA2a, collectively ensuring ROS homeostasis and improved osmoregulation [113]. SiCDPK27 functions through phosphorylating HY5 to facilitate its cold-induced accumulation and the subsequent upregulation of flavonoid levels [114]. AtCDPK1 regulates plant thermotolerance through the ABA signaling pathway [115,116]. Under heat stress, ZmCDPK7 phosphorylates SHSP17.4 to maintain protein quality. Conversely, its phosphorylation of RBOHB modulates ROS accumulation [117]. SlCDPK28 enhances plant thermotolerance by phosphorylating SlAPX2 to modulate the cellular redox homeostasis [118]. DoCDPK20 enhances plant heat tolerance by regulating peroxidase activity to decrease ROS accumulation [119]. By www.figdraw.com (accessed on 9 December 2025).

Temperature shapes plant geographical distribution and profoundly influences their growth, survival, and productivity. Chilling stress prevents crops from attaining their full yield capacity, impacting all developmental phases from germination to fruit formation [108]. Cold stress triggers a suite of physiological and morphological adaptations in plants, including alterations in the cell membrane and chloroplast structures, and modulate cold signaling pathways involving plant hormones, reactive oxygen species (ROS), protein kinases, and inorganic ions. These adaptations are generally achieved through extensive transcriptional regulation [109,110]. CDPK, as a crucial signaling mediator, has been extensively studied in this regard (Figure 4).

In rice, CDPKs regulate cold tolerance through multiple signaling pathways. OsCDPK17 phosphorylates and stimulates the aquaporin OsPIP2;1, OsPIP2;6. This regulation enhances cellular osmotic adjustment and improves water permeability within guard cells, facilitating prompt stomatal closure under low temperatures. Additionally, OsCDPK17 appears to influence broader metabolic processes by suppressing the activity of certain elements in sugar and nitrogen assimilation pathways. This inhibitory influence on metabolic activity likely enables plants to redirect resources to essential cold adaptation processes, thereby improving freezing resilience [111]. The overexpression of *OsCDPK24* enhances cold tolerance in plants. Further functional analysis revealed that *OsCDPK24* phosphorylates *OsGrx10*, a thioltransferase whose activity is reduced upon interaction with *OsCDPK24*. This decrease in activity limits glutathione (GSH) consumption, leading to elevated GSH levels and an increased GSH/GSSG (reduced glutathione to oxidized glutathione) ratio, thereby reducing oxidative injury induced by low temperatures. OsCDPK24 phosphorylates another known substrate, OsANN1, a membrane-associated annexin that confers oxidative stress protection via elevated activities of the anti-oxidative enzymes superoxide dismutase and catalase, ultimately improving stress tolerance [112,120,121]. Its homolog in maize, ZmCDPK1, is activated in low-temperature environments. However, heterologous expression of ZmCDPK1 in Arabidopsis led to decreased cold resilience in transgenic lines, pointing to its putative role as a repressor of cold stress signaling [122]. In orchids, the expression of *PaCDPK1* is induced at the transcriptional level under low-temperature stress; however, its precise functional roles remain to be further elucidated [123]. Indeed, in addition to rice, CDPKs reportedly increase cold tolerance by facilitating ROS scavenging in various plant species. In peach, PpCDPK29 associates with respiratory burst oxidase homolog protein (PpRBOHC/D) and antioxidant enzymes (PpSOD and PpCAT1) thereby ensuring the maintenance of ROS homeostasis. Furthermore, PpCDPK29 phosphorylates PpHSFA2a, increasing its DNA-binding affinity for target gene promoters. This phosphorylation event enables PpHSFA2a to activate the transcription of key stress-responsive genes, including *PpHSP18.5*, *PpHSP70*, *PpGSTU7*, *PpGSTU19*, *PpGolS1*, and *PpBAM1*. The encoded proteins function as molecular chaperones or contribute to ROS scavenging and osmotic regulation, collectively alleviating chilling injury in postharvest peach fruit [113]. In apple (*Malus domestica*), heterologous expression of *MdCDPK1a* in tobacco improves freezing resilience by reducing ROS levels and preserving cellular equilibrium [124]. Similarly, heterologous expression of *SikCDPK1* in transgenic tobacco plants via overexpression constructs resulted in increased cold tolerance. Transcriptomic analysis further revealed that SikCDPK1 positively regulates low-temperature adaptation, partly through decreasing ROS levels [125,126]. In tomato (*Solanum lycopersicum*), cold stress upregulates the transcription of *SiCDPK27* and promotes ABA biosynthesis. Silencing of *SiCDPK27* was found to reduce the cold-induced accumulation of NO and H_2_O_2_, suppress MPK1/2 activation under ABA signaling, and decrease cold tolerance. Further investigation by Lin et al. (2025) revealed that SiCDPK27 phosphorylates the HY5 (ELONGATED HYPOCOTYL5) protein under cold stress. HY5, in turn, binds directly to promoter regions to activate the transcription of flavonoid biosynthetic genes and modulates both the CBF regulatory pathway and flavonoid biosynthesis, thereby enhancing cold acclimation in tomato [114,127].

### 4.4. CDPK Under Heat Stress

Global warming represents one of the most urgent climatic threats confronting contemporary society. Agricultural production is increasingly threatened by climate change, Climate change poses a growing threat to agricultural production, with persistently rising temperatures exerting detrimental effects on major crops such as wheat and rice [128,129,130]. Enhancing plant thermotolerance must therefore become a central focus of scientific research. Heat stress refers to an increase in ambient temperature of 10–15 °C, which typically causes heat injury across molecular, cellular, tissue, organ, organismal levels. Elevated temperatures during seed germination can suppress or entirely arrest this biological process. At later growth stages, heat stress adversely affects photosynthesis, respiration, water balance, and membrane integrity. When plants undergo thermal stress throughout their growth phase, it not only alters phytohormone levels and various primary and secondary metabolites but also elicits molecular reactions, including heat shock protein induction, the synthesis of additional stress-associated proteins [66,131,132,133]. To alleviate the adverse effects of elevated temperatures, plants have developed complex signaling systems that detect increases in environmental heat and initiate protective mechanisms via transcriptional, protein-level, and metabolic reprogramming [134,135,136]. These adaptations include maintaining protein stability, scavenging harmful ROS, activating relevant signal transduction pathways such as the MAPK and CDPK cascades, and accumulating and modulating compatible solutes to sustain normal growth under heat stress (Figure 4) [137,138,139].

Multiple studies have reported that *AtCDPK1* positively regulates both thermotolerance and cold tolerance in plants. Veremeichik et al. (2025) demonstrated that the overexpression of either the native *AtCDPK1* or a constitutively active form (AtCDPK1-Ca), in which its autoinhibitory domain is inactivated, enhances heat tolerance in *R. cordifolia* L. calli. Research in tobacco further revealed that under nonstress conditions, constitutively active *AtCDPK1* stimulates ABA biosynthesis. However, under high-temperature conditions, ABA levels decreased in the transgenic plants, indicating that AtCDPK1 could regulate stress adaptation by influencing abscisic acid and salicylic acid signaling [115,116]. Using iTRAQ-based quantitative proteomics, Researcher reported that ZmCDPK7 functions as a thermally responsive kinase in maize. The overexpression of *ZmCDPK7* was shown to increase thermotolerance in transgenic plants. This improvement was attributed to the elevated activities of CAT and APX, which significantly alleviated thermal injury to membranes and photosynthetic structures, thereby reducing ROS accumulation in maize under heat stress. Heat shock proteins (HSPs) function as molecular chaperones critical for thermotolerance, facilitating the refolding of denatured proteins under heat stress. ZmCDPK7 phosphorylates sHSP17.4, thereby boosting its chaperone capacity, consequently mitigating injury to cellular membranes and photosynthetic components during heat stress [117,140,141]. In tomato, *SlCDPK28* is a positive regulator of thermotolerance. Knockout of SlCDPK28 significantly reduces heat tolerance, while SlCDPK28 phosphorylates the ascorbate peroxidase SlAPX2; this regulatory pathway modulates cellular redox homeostasis, thereby increasing plant thermotolerance [118]. In yam (*Dioscorea opposita*), the expression of *DoCDPK20* is significantly induced under high-temperature conditions. Overexpression of *DoCDPK20* alleviates heat-induced damage to photosynthesis in transgenic tobacco plants. These transgenic lines presented improved photosynthetic efficiency and enhanced stress resilience, along with markedly elevated activities of SOD, CAT, and POD; decreased ROS levels; and better cellular equilibrium, together promoting heat tolerance [119].

### 4.5. CDPK Under Trace Metal Stress

In recent decades, the rapid pace of urbanization and industrial growth has led to a significant increase in trace metal pollution, causing considerable harm to the environment and ecosystems [118,142,143]. The elevated trace metals in the soil and atmosphere are directly transferred to plants, negatively impacting their development and potentially posing health threats to end consumers, such as animals and humans [118,142,143,144,145]. Trace metals are found in the Earth’s crust and soils at naturally low abundance levels. These elements include copper (Cu), iron (Fe), zinc (Zn), nickel (Ni), cobalt (Co), aluminum (Al), cadmium (Cd), chromium (Cr), lead (Pb), arsenic (As), and mercury (Hg). Among these, certain metals, such as Fe, manganese (Mn), Zn, copper (Cu), and molybdenum (Mo), are essential or beneficial for plant metabolism and growth. However, when these compounds accumulate at elevated concentrations, they can become toxic to plants. In contrast, other trace metals and metalloids, such as Cd, Hg, As, Pb, and Cr, are highly phytotoxic even at low concentrations [146,147]. When plants are subjected to metal toxicity, numerous signaling molecules, including hormones, ROS, Ca^2+^ ions, MAPKs, and nitric oxide (NO), trigger signal transduction pathways to activate defense mechanisms [84,148,149]. Moreover, CDPKs are not only involved in maintaining the homeostasis of certain essential trace metals but also contribute to tolerance to several toxic elements.

Copper (Cu) serves as a vital micronutrient necessary for proper plant growth and development. It performs an essential function in photosynthetic performance and serves as a key component of various oxidases, thereby influencing redox reactions and respiratory processes in plants. Furthermore, copper is extensively involved in protein trafficking and supports the preservation of cellular membrane integrity [150]. Under copper-deficient conditions, plants exhibit retarded growth and may even exhibit impaired flowering and pollination processes. Additionally, leaf malformation and reduced crop yield are common [151,152]. However, excessive copper also has detrimental effects on plants, including impaired photosynthetic function, disrupted growth and development, and perturbs the uptake and accumulation of other essential mineral nutrients [153]. In the marine alga *Ulva compressa*, CDPKs are involved in tolerance and detoxification mechanisms under excess copper. Exposure of *U. compressa* to 10 μM copper for 24 h directly induced an increase in NO levels, accompanied by increased nitric oxide synthase (NOS) activity. This is followed by the activation of calcium channels, leading to elevated cytosolic Ca^2+^ concentrations, and the upregulation of expression of genes encoding antioxidant enzymes, as well as those involved in ascorbate (ASC) and GSH biosynthesis, as well as metallothioneins (MTs). This entire process is regulated through the activation of calcium-sensing proteins such as calmodulins (CaMs) and CDPKs, as well as the oxidative stress-dependent MAPKK kinase MEK1/2 [154,155].

Manganese (Mn), a vital micronutrient for plants, is involved in diverse metabolic pathways, including photosynthesis, respiration, and fatty acid and protein synthesis, and functions as an activator or cofactor for numerous enzymes. Although manganese provides numerous benefits for plant growth, excessive uptake can result in toxicity and adversely affect plant development. Zhang et al. (2021) revealed that alterations in manganese levels influence calcium ion signaling. Specifically, high-manganese treatment of *A. thaliana* induced an increase in the cytosolic calcium concentration. Furthermore, AtCDPK4/5/6/11 interact with the manganese transporter MTP8, positively regulating manganese stress tolerance and playing a crucial role in maintaining manganese homeostasis [156]. A year later, their research team revealed a new pathway, CBL2/3–CIPK3/9/26–MTP8, through which the calcium sensors CBL2/3 recruit the kinases CIPK3/9/26 to form a complex that primarily phosphorylates MTP8 at Ser35, thereby negatively regulating its transport activity from the cytoplasm to the vacuole. In contrast, CDPKs primarily target the N-terminal region of MTP8 for phosphorylation at the Ser31 and Ser32 sites, leading to its activation and facilitating the sequestration of excess Mn^2+^ into the vacuole. These two calcium-dependent regulatory mechanisms collectively modulate manganese homeostasis in response to fluctuating manganese concentrations [157].

Iron (Fe) is a vital micronutrient for plants and is integral to key physiological processes, including respiration, photosynthesis, and antioxidant defense, as well as numerous biochemical pathways. Both iron deficiency and excess can lead to severe metabolic disorders, adversely affecting respiratory and photosynthetic functions as well as overall plant health, thereby ultimately compromising crop productivity [158,159]. Plants primarily acquire iron through their root systems from the soil. Although iron is abundant in most soils, under neutral pH conditions, it predominantly exists in the form of insoluble complexes, such as ferric (Fe^3+^) hydroxides, rendering it largely unavailable for plant uptake. Consequently, plants from different families have evolved two distinct strategies for iron acquisition. Graminaceous plants, such as rice, employ a “chelation strategy” for iron acquisition, which involves the secretion of phytosiderophores (PSs). These phytosiderophores are released into the soil via transporters of the transporter of mugineic acid (TOM) family. The PS molecules form stable complexes with Fe(III), and the resulting Fe(III)–PS complexes are subsequently taken up into root cells by members of the YELLOW STRIPE-LIKE (YSL) transporter family. Nongraminaceous species employ a “reduction strategy” for iron uptake. This mechanism initiates rhizosphere acidification via plasma membrane H^+^-ATPase activity, thereby improving ferric iron [Fe(III)] solubility. Subsequently, membrane-associated ferric chelate reductases catalyze the reduction of Fe(III) to the ferrous form [Fe(II)], making it available for uptake. In *Arabidopsis thaliana*, the iron-regulated transporter AtIRT1 is primarily responsible for the influx of Fe(II) from the soil into root epidermal cells, thereby completing the iron acquisition process [159,160,161,162]. Under iron-deficient conditions, IRT1 cannot transport sufficient iron, thereby adversely affecting plant growth. In *A. thaliana*, the transcription of AtCDPK21 and AtCDPK23 is increased under iron-deficient conditions. The double mutant cpk21/cpk23 exhibited heightened sensitivity to iron limitation. Further mechanistic studies revealed that CDPK21 and CDPK23 phosphorylate IRT1 at Ser149, which modulates transporter activity, contributes to cellular iron homeostasis, and positively regulates plant tolerance to iron deficiency [163].

Boron (B) is an essential micronutrient indispensable for plant structure and metabolism, with key functions in processes such as cell wall biosynthesis, sugar transport, and enzyme modulation [164]. AtBOR1, a boron efflux transporter, mediates the translocation of boron from roots to the xylem under boron limitation [165]. Through BiFC assays, researchers identified several CDPK proteins that potentially interact with BOR1 and participate in boron transport. Phenotypic analysis of mutant lines further revealed CDPK10 as a candidate gene associated with sensitivity to boron deficiency. Further analysis revealed that CDPK10 associates with BOR1 and phosphorylates it at Ser689, enhancing its transporter activity. This modification facilitates boron translocation from roots to the xylem, ultimately improving plant tolerance to boron deficiency [166].

Arsenic (As), a known human carcinogen, is a nonessential element for plant growth. However, plants can absorb arsenate from the environment through phosphate transporters, leading to its accumulation in plant tissues. When soil and water are contaminated, arsenic may be transferred into crops, ultimately posing serious risks to human health [167]. Under arsenic (As) treatment, rice roots exhibit calcium oscillations, accompanied by the upregulation of multiple calcium sensor genes. These include seven calmodulin, two CBL, one CIPK (OsCIPK21), and four CDPK (OsCDPK4/13/20/21) genes. Calcium signaling pathways are implicated by these observations in the plant’s adaptation to arsenate [As(V)] toxicity [168]. Researchers identified AtCDPK31 as an interacting partner of the As(III) transporter AtNIP1 via Y2H and BiFC assays. *cpk31* T-DNA insertion knockout lines consistently presented increased tolerance to As(III). Notably, the cpk31-1 mutant presented markedly lower arsenic accumulation rates in both the root and shoot tissues than did the wild-type plants [169]. Researchers identified the *cpk23* mutant of *Arabidopsis* as exhibiting the most sensitive phenotype under arsenate [As(V)] stress through phenotypic screening. However, neither the arsenic tolerance nor the transcriptional level of *CDPK23* was increased in the *CDPK23*-overexpressing lines upon As(V) treatment, suggesting that the regulation of CDPK23 by arsenic may occur at the kinase activity level rather than through transcriptional activation. To test this hypothesis, a constitutively active form of CDPK23 (CDPK23-VK) was generated. Transgenic plants expressing CDPK23-VK presented significantly increased tolerance to As(V) stress, confirming the importance of posttranslational activation of CDPK23 in mediating As resistance. Using immunoprecipitation–mass spectrometry (IP-MS), researchers screened for proteins that potentially interact with CDPK23 and identified PHT1;1, a PHT1 family phosphate transporter that also facilitates arsenate [As(V)] uptake. Further analysis demonstrated that CDPK23 phosphorylates PHT1;1, thereby modulating arsenic uptake and specifically enhancing plant tolerance to As(V) [170,171,172].

Cadmium (Cd), a heavy metal pollutant, infiltrates water and soil and subsequently accumulates in crops. Both natural and anthropogenic processes contribute to the substantial release of cadmium into the environment [173]. Through long-term natural selection, many plant species have evolved cadmium tolerance. For example, *Sedum alfredii* Hance can accumulate more than 7000 μg of Cd per gram of dry weight [174]. Cadmium is highly mobile and bioavailable, facilitating its uptake by plant roots and subsequent accumulation within tissues. As a nonrequired element, cadmium disrupts metabolic and biochemical pathways by replacing essential metal ions and interacting with functional groups in biomolecules, ultimately leading to impaired plant growth and reduced productivity [175,176,177]. Although no specific cadmium transport proteins exist in plants, cadmium can be erroneously recognized and transported due to its structural similarity to essential nutrient elements such as Fe, Zn, Ca, and Mn. Proteins of the natural resistance-associated macrophage protein (NRAMP) family, which are transmembrane transporters involved in metal ion homeostasis, have been demonstrated to facilitate the transport of cadmium in multiple species [178,179,180]. High concentrations of cadmium activate calcium signaling pathways. Researchers have discovered that AtCDPK21/23 interact with the cadmium transporter NRAMP6. Cadmium stress upregulates the expression of both CDPK21 and CDPK23. Phenotypic analysis of the corresponding mutants revealed that CDPK21 and CDPK23 are involved in the cadmium stress response. Specifically, CDPK21 and CDPK23 phosphorylate NRAMP6, thereby inhibiting its transport activity and positively regulating cadmium tolerance in *Arabidopsis* [178,181]. The expression of *PeCDPK21* in poplar is activated upon Cd stress. Overexpression of *PeCDPK21* enhances Cd tolerance in *Arabidopsis*. Using HaloTag-based pull-down assays followed by expression profiling, researchers identified multiple proteins associated with the heavy metal stress response. These findings suggest that *PeCDPK21* may interact with cation/heavy metal transporters to modulate Cd translocation, cooperate with antioxidant enzymes to maintain ROS homeostasis under Cd stress, and engage with integral membrane proteins to regulate water status, thereby positively regulating Cd tolerance. Furthermore, *PeCDPK21* interacts with the transcription factor AtNF-YC3, a known positive regulator of cadmium tolerance in *Arabidopsis*. Their interaction contributes to reduced Cd uptake and enhanced ROS scavenging in transgenic plants [182,183].

## 5. CDPKs: Key Mediators Bridging Plant Innate Immunity and Defense-Related Molecular Networks

Plants have evolved a sophisticated innate immune system to defend against diverse environmental microorganisms. This system relies on cellular receptors that, upon recognition of invasion signals, initiate pattern-triggered immunity (PTI) or effector-triggered immunity (ETI) [184]. PTI represents a broad-spectrum and nonspecific defense response in plants, providing basal resistance against microbial pathogens. Pattern recognition receptors (PRRs) identify conserved molecular structures from both phylogenetically related microbes and endogenous damage signals. These structures include bacterial flagellin (flg22) and elongation factor Tu (EF-Tu), endogenous AtPep peptides, and chitin oligomers from fungi and bacterial peptidoglycan, which are recognized by lysin motif (LysM)-containing receptor-like kinases such as CERK1 [185,186]. However, certain pathogens express virulence factors, known as effectors, which evade recognition by PRRs. These effectors modulate plant immunity to promote infection. In response, plants have developed intracellular immune receptors capable of directly or indirectly detecting these effectors, forming an additional defense layer known as ETI. This receptor class primarily consists of proteins bearing nucleotide-binding (NB) and leucine-rich repeat (LRR) domains, known as NLRs. NLR activation culminates in a potent defense response, often characterized by a hypersensitive response (HR) involving localized cell death, which is a hallmark of ETI [187]. CDPK has been demonstrated to play critical and conserved roles in plant immune responses across multiple plant species, including *Arabidopsis thaliana*, rice, potato, and wheat [188,189,190,191,192].

Among the numerous members of the CDPK family, CDPK5 has emerged as a central target in plant immunity research because of its involvement in multiple immune regulatory pathways. CDPK5 activates defense responses primarily through the phosphorylation of downstream target proteins, which can be categorized into three major aspects on the basis of current findings: In the context of PAMP signals or ROS stimulation, CDPK5 phosphorylates and activates respiratory burst oxidase homolog D (RBOHD), thereby promoting ROS production [193]. Moreover, by phosphorylating and activating WRKY transcription factors, CDPK5 orchestrates the induction of key defense-related compounds, including salicylic acid (SA), ethylene, and camalexin [194]. CDPK5 also participates in defense mechanisms activated by oligogalacturonides (OGs), which are oligomers of α-1,4-linked galacturonic acid residues and function as typical DAMPs. Mutations in the *CDPK5*, *CDPK6*, and *CDPK11* genes increase the susceptibility of *Arabidopsis* to *Botrytis cinerea* and lead to a loss of OG-induced immunity [195,196]. In addition to its direct phosphorylation of downstream target proteins, the activity of CDPK5 is subject to regulation by other immune components. A notable example is TN2, a truncated NLR protein that plays a key role in modulating CDPK5 function. TN2 interacts with CDPK5 and locks it in its activated, open conformation. This interaction enhances and sustains CDPK5 kinase activity, thereby amplifying downstream defense signaling cascades [197]. Moreover, CDPK5 indirectly promotes the expression of defense-related genes by modulating transcription factors and transcriptional regulatory complexes. Two central pathways involved in this process are the CDPK5–CBP60g and the CDPK5–MORC1–NPR1–TGA modules. CBP60g is a calmodulin-binding protein in plants that functions as a master transcriptional regulator whose expression is induced upon pathogen infection, leading to the activation of a broad array of defense-related genes. CDPK4, CDPK5, CDPK6, and CDPK11 directly phosphorylate CBP60g, thereby increasing its DNA-binding activity and consequently increasing plant resistance to *Verticillium dahliae* [198,199]. MICRORCHIDIA (MORC) proteins are members of an evolutionarily conserved GHKL-type ATPase superfamily. MORC1, also known as TCV 1-IMPAIRED (TCV-INCOMPATIBLE) INTERACTION 1 (CRT1), is involved in various aspects of plant immune responses, including both PTI and ETI. CDPK5 binds directly to the N-terminal region of MORC1 and mediates its phosphorylation. The phosphorylation event stabilizes MORC1 and drives its translocation into the nucleus. In addition, nucleus-localized MORC1 interacts with the TGA/NPR1 transcriptional complex to stimulate defense-related gene expression and increase disease resistance in *Arabidopsis* [200,201,202]. Indeed, across diverse plant species, CDPKs mediate responses to biotic stress through interactions with WRKY transcription factors, highlighting an evolutionarily conserved regulatory module in plant immunity. In rice, the transcription factor OsWRKY45-1 contributes significantly to resistance against fungal and bacterial pathogens. TaCDPK2-A, a wheat CDPK, can regulate the expression of OsWRKY45-1 in transgenic rice and VIGS-treated wheat plants [203]. AtCDPK4/5/6/11 phosphorylate a specific subset of WRKY transcription factors (WRKY8/28/48), which regulate key transcriptional reprogramming events that restrict pathogen growth. In pepper, CaWRKY40 functions as a promoter during *Ralstonia solanacearum* infection (RSI) [204]. The expression of CaCDPK15 is upregulated upon RSI, and CaCDPK15 regulates the expression of CaWRKY40. Unlike the direct phosphorylation mechanisms observed in other systems, the interaction between CaCDPK15 and CaWRKY40 is indirect. Furthermore, CaWRKY40 specifically associates with the promoter region of CaCDPK15 and induces its transcriptional activation, forming a positive feedback loop that amplifies the immune signal [205]. In addition to positively regulating disease resistance, certain CDPKs are also involved in the suppression of plant immune responses. For example, CDPK28 acts as a negative immune regulator through two distinct pathways during biotic stress responses. On the one hand, CDPK28 directly phosphorylates the E3 ubiquitin ligases PUB25/26, which in turn promotes the degradation of BIK1 through ubiquitination, thereby suppressing immune signaling [206,207,208]. On the other hand, CDPK28 phosphorylates the aquaporin PIP2;7, which normally facilitates H_2_O_2_ transport and positively regulates immunity. This leads to PIP2;7 protein degradation and impaired function. Notably, both processes are subject to precise balancing mechanisms. PAMP perception induces the expression of the ubiquitin ligases ATL6 and ATL31, which ubiquitinate CDPK28 and target it for proteasomal degradation, thereby promoting BIK1 stability. Furthermore, the interaction between CDPK28 and PIP2;7 is inherently unstable and dissociates upon pathogen infection, providing an additional layer of regulatory control [209,210].

## 6. CDPK in Phytohormone Signaling Pathways

CDPKs participate extensively in the synthesis and signal transduction of diverse phytohormones. CDPK4 and CDPK11 serve as positive regulators of ABA signaling via the CDPK/calcium pathway in *Arabidopsis thaliana*. These kinases modulate ABA signal transduction and directly phosphorylate AtIpk2β (an *Arabidopsis* inositol polyphosphate kinase), thereby enhancing ABA-responsive signaling. CDPK11 also initiates a transcriptional cascade by activating ABF/AREB/ABI5 clade bZIP factors, which subsequently bind ABA-responsive elements and modulate downstream gene expression [211,212,213]. AtCDPK32 has been shown to interact with AtABF4 in vitro, and overexpression of *AtCDPK32* results in ABA-hypersensitive phenotypes. CDPK32 participates in ABA signal transduction by modulating the activity of ABF/AREB (ABA-responsive element-binding factor) transcription factors [214].

Gibberellic acid (GA) signaling responses, transcriptional regulation, and vacuolar function modulation involve the participation of CDPKs, with species-specific mechanisms underlying their functions across diverse plant species. In *Arabidopsis thaliana*, AtCDPK28 functions as a positive regulator of GA homeostasis [215]. In potato (*Solanum tuberosum*), the expression of *StCDPK1* is upregulated within two hours of GA_3_ treatment under tuber-inducing conditions, accompanied by a gradual increase in the expression of GA20ox. This identifies it as a key GA biosynthetic enzyme, pointing to its potential role in the feedback regulatory loop controlling GA homeostasis [216,217]. StCDPK3 can phosphorylate StRSG1, a transcription factor related to RSG, in vitro, indicating its potential role in GA signaling [218]. In tobacco (*Nicotiana tabacum*), NtCDPK1 negatively regulates GA homeostasis by suppressing the activity of RSG, a bZIP transcription factor. GA activates NtCDPK1, which phosphorylates NtRSG, facilitating its interaction with 14-3-3 proteins and leading to nuclear export, thereby inhibiting the transcription of *GA20ox*, a critical GA biosynthetic enzyme [219]. Sucrose deficiency and GA selectively induce OsCDPK1 expression during early seedling development in rice. It negatively regulates the expression of GA biosynthetic genes (*GA20ox1*, *GA3ox2*) and upregulates the 14-3-3 protein-encoding gene *GF14c*, thereby reducing GA biosynthesis [220]. In barley (*Hordeum vulgare*), HvCDPK1 regulates GA responses in barley aleurone cells through the modulation of vacuolar activity, thereby influencing physiological processes in the aleurone layer [221].

CDPKs modulate ethylene biosynthesis by phosphorylating ACC synthase (ACS), the rate-limiting enzyme in ethylene production, although their functional outcomes vary across species [222]. In *Arabidopsis thaliana*, AtCDPK16 phosphorylates AtACS7 to regulate ethylene levels. Similarly, CDPK5 and CDPK6 contribute to wound-induced ethylene synthesis by modulating the expression of *ACS* genes [223,224]. In tomato, LeCDPK2 phosphorylates LeACS2. Its expression is induced by multiple signals, suggesting dual roles in both ethylene biosynthesis and the signal response [225,226]. In cucumber (*Cucumis sativus*), CsCDPK6 interacts with CsSAMS1, a key enzyme in the methionine cycle, thereby influencing the balance between polyamine and ethylene biosynthesis. The overexpression of *CsCDPK6* in tobacco enhances salt tolerance, highlighting its potential role in stress adaptation [107].

CDPKs regulate auxin-related physiological processes through multiple mechanisms, including phosphorylation of auxin transporters, participation in auxin response pathways, and transcriptional or posttranslational modulation by auxin signaling. In potato, StCDPK1 may contribute to tuber formation by phosphorylating StPIN proteins to modulate auxin transport, a process potentially interconnected with gibberellin (GA) biosynthesis [227]. AtCDPK29 specifically targets unique phosphorylation sites in PIN-HLs, namely, Ser253 in PIN1 and Ser259 in PIN2. CDPK29 is essential for PIN-mediated developmental processes [228]. Additionally, AtCDPK3 and AtCDPK4 phosphorylate AtPLA IVA and IVB (patatin-like phospholipase A). Since pharmacological inhibition of PLAs suppresses the expression of *Auxin/IAA* genes and the auxin-activated *DR5* promoter, CDPKs may regulate auxin signaling through PLA-mediated pathways [229,230]. Some CDPKs are also modulated by auxin. For example, the expression of *MsCDPK3* in alfalfa (*Medicago sativa*) and the activity of a 50 kDa CDPK in cucumber are influenced by auxin, although the precise regulatory mechanisms involved remain to be elucidated [231,232].

CDPKs play pivotal and diverse roles in the biosynthesis and signaling of JA, with functional variation often observed among orthologs across species, including both synergistic and antagonistic effects. On the one hand, JA can modulate the activity and expression of CDPKs. For example, in maize, *ZmCDPK11* is regulated by linolenic acid (LA) and methyl jasmonate (MeJA). In tobacco, *NtCDPK1* and other CDPKs are induced by MeJA, whereas in potato, *StCDPK2* is downregulated by JA [233,234,235]. On the other hand, CDPKs positively and negatively regulate JA pathways. In *Nicotiana plants*, simultaneous silencing of *NaCDPK4* and *NaCDPK5* leads to a significant increase in the levels of OPDA (12-oxo-phytodienoic acid), a key precursor in JA biosynthesis [236,237,238]. Additionally, certain CDPKs contribute to JA homeostasis, and JA signaling involves crosstalk with MAPK cascades [238]. For instance, AtCDPK28 modulates JA and GA homeostasis to achieve an optimal balance between growth and defense [32,215].

## 7. Conclusions

In the face of increasing environmental challenges, including rising temperatures, droughts, floods, and soil pollution, plant growth and productivity are increasingly threatened [239,240,241,242]. Understanding the mechanisms underlying plant stress resistance, as well as the trade-offs between growth and defense, has thus become a critical research priority. Ca^2+^ serves as an essential secondary messenger in plant stress responses and developmental processes [243]. Among the specific calcium sensors in plants, CDPKs play a central role in decoding Ca^2+^ signals and transducing them into appropriate physiological outputs [244]. Current research has extensively characterized the functions of CDPKs, revealing their roles as versatile signaling nodes that often exhibit pleiotropic effects and participate in multiple regulatory networks influencing both plant growth and defense responses [245]. Current studies report numerous CDPK genes. These genes can be implicated in regulating distinct pathways. They affect plant growth and stress resistance in different or even opposing directions. Whether this process involves cell-type-specific signaling remains an open question worthy of discussion. While numerous CDPK-mediated substrates and target proteins have been identified, the molecular mechanisms underlying most CDPKs remain incompletely understood. CDPK regulation is complex, especially its involvement in overlapping and sometimes conflicting pathways. Therefore, several unresolved questions remain. These include the nature of the crosstalk between calcium signals, CDPKs, and other signaling molecules; the potential interconnectivity among pathways regulated by a single CDPK; the occurrence of synergistic or antagonistic interactions between different CDPKs within the same pathway; and the precise mechanisms controlling CDPK activity itself. In recent years, Liese et al. (2023) have developed a Förster resonance energy transfer (FRET)-based genetically encoded reporter that visualizes calcium-dependent protein kinase (CDPK) conformational changes, thereby enabling real-time decoding of Ca^2+^ signals in plants [246]. This technology provides a critical tool for decoding calcium signaling by CDPKs at the cellular level. Future efforts to correlate these real-time CDPK conformational dynamics with specific outputs in plant growth and stress response signaling would significantly advance our mechanistic understanding of these processes.

## Data Availability

No new data were created or analyzed in this study. Data sharing is not applicable.
